# Rapid MALDI-MS Assays for Drug Quantification in Biological Matrices: Lessons Learned, New Developments, and Future Perspectives

**DOI:** 10.3390/molecules26051281

**Published:** 2021-02-26

**Authors:** Margaux Fresnais, Esra Yildirim, Seda Karabulut, Dirk Jäger, Inka Zörnig, Julia Benzel, Kristian W. Pajtler, Stefan M. Pfister, Jürgen Burhenne, Walter E. Haefeli, Rémi Longuespée

**Affiliations:** 1 Department of Clinical Pharmacology and Pharmacoepidemiology, Heidelberg University Hospital, Im Neuenheimer Feld 410, 69120 Heidelberg, Germany; Margaux.Fresnais@med.uni-heidelberg.de (M.F.); esra.yildirim@med.uni-heidelberg.de (E.Y.); sedaaka4155@gmail.com (S.K.); juergen.burhenne@med.uni-heidelberg.de (J.B.); Walter-Emil.Haefeli@med.uni-heidelberg.de (W.E.H.); 2 National Center for Tumor Diseases Heidelberg, Department of Medical Oncology, Heidelberg University Hospital, Im Neuenheimer Feld 460, 69120 Heidelberg, Germany; dirk.jaeger@nct-heidelberg.de (D.J.); inka.zoernig@nct-heidelberg.de (I.Z.); 3 Hopp Children’s Cancer Center Heidelberg (KiTZ), Im Neuenheimer Feld 430, 69120 Heidelberg, Germany; j.benzel@kitz-heidelberg.de (J.B.); k.pajtler@kitz-heidelberg.de (K.W.P.); s.pfister@kitz-heidelberg.de (S.M.P.); 4 German Cancer Research Center (DKFZ), Division of Pediatric Neurooncology, German Cancer Consortium (DKTK), Im Neuenheimer Feld 280, 69120 Heidelberg, Germany; 5 Department of Pediatric Hematology, Oncology and Immunology, Heidelberg University Hospital, 69120 Heidelberg, Germany

**Keywords:** mass spectrometry, MALDI, therapeutic drug monitoring, targeted quantification

## Abstract

Matrix-assisted laser desorption/ionization mass spectrometry (MALDI-MS) has rarely been used in the field of therapeutic drug monitoring, partly because of the complexity of the ionization processes between the compounds to be quantified and the many MALDI matrices available. The development of a viable MALDI-MS method that meets regulatory guidelines for bioanalytical method validation requires prior knowledge of the suitability of (i) the MALDI matrix with the analyte class and properties for ionization, (ii) the crystallization properties of the MALDI matrix with automation features, and (iii) the MS instrumentation used to achieve sensitive and specific measurements in order to determine low pharmacological drug concentrations in biological matrices. In the present hybrid article/white paper, we review the developments required for the establishment of MALDI-MS assays for the quantification of drugs in tissues and plasma, illustrated with concrete results for the different steps. We summarize the necessary parameters that need to be controlled for the successful development of fully validated MALDI-MS methods according to regulatory authorities, as well as currently unsolved problems and promising ways to address them. Finally, we propose an expert opinion on future perspectives and needs in order to establish MALDI-MS as a universal method for therapeutic drug monitoring.

## 1. Introduction

Liquid chromatography coupled to electrospray ionization-tandem mass spectrometry (LC-ESI-MS/MS) has become the gold standard for the quantification of most drugs in diverse biological matrices. LC allows for the separation of drugs from endogenous compounds co-extracted from the biological matrix. ESI is a widely described ion source for drug quantification, which relies on well-described electrochemical processes [1]. For this reason, LC-ESI-MS/MS does not require an extensive knowledge of complex ionization processes before routine use for diverse applications. Methods for MS analyses of surfaces are progressively emerging as options for drug quantification from biological matrices [2,3]. Matrix-assisted laser desorption/ionization (MALDI) uses laser energy-absorbing organic compounds—called the MALDI matrix—for the ionization of small to large molecules. The myriad of MALDI matrices for diverse classes of small compounds, and their availability with diverse solvent mixtures for their dissolution and downstream proper crystallization [4], do not allow for a straightforward use of MALDI without extensive expertise. For drug analysis, the experiment often prevails over the prediction of the ionization of a compound of interest for the determination of a suitable MALDI matrix. The various and heterogeneous crystal morphologies of MALDI matrices also lead to adapted developments in order to enable automated analyses. For instance, 2,5-dihydroxybenzoic acid (2,5-DHB) is one of the most versatile MALDI matrices for the analysis of small compound drugs, but its crystallization is highly heterogeneous when manual deposition is used. This crystal heterogeneity makes it difficult to obtain reproducible signals when using automated acquisition. Finally, the absence of upstream chromatographic or electrophoretic separation often forces the user to analyze complex multi-compound mixtures. Therefore, adapted MS instrumentations and features, as well as analytical workarounds, are required to perform specific—and therefore sensitive—analyses. For all these reasons, MALDI has not been widely used as an MS method to quantify drugs. However, besides its drawbacks, some characteristics of MALDI-MS for drug analyses offer extremely valuable advantages compared to LC-ESI-MS/MS assays. The most important advantage is the rapidity of workflows from sample preparation to final quantification that can be achieved using MALDI-MS. Although this is a disadvantage for further analytical specificity, the absence of chromatographic or electrophoretic separation theoretically allows analyses of any complex mixture dissolved in any solvent to be performed. Therefore, shorter and simpler sample preparation is possible prior to MALDI-MS analyses. In addition, MALDI-MS drug analysis in tissues or plasma usually requires only small sample volumes. Finally, when MALDI-MS is used in imaging mode, it confers the possibility of quantifying drugs in a given histological context.

Currently, only a few studies have reported the establishment of MALDI-MS assays for drug quantification in plasma, such as human immunodeficiency virus (HIV) protease inhibitors [5], the CIGB-300 antitumor peptide [6], methotrexate [7], pemetrexed [8], and osimertinib (OSN) [9]. In tissue sections, MALDI-MS has been used for a wider panel of applications for drug quantification, but mostly in the context of MALDI imaging investigations [10]. Our group recently developed rapid MALDI-MS assays for the quantification of mebendazole (MBZ) in tissue sections [11] and OSN [9] in plasma, with the last method being fully validated according to regulatory authorities (US Food and Drug administration (FDA) and European Medicine Agency (EMA)) [12,13]. Series of tests for method development have provided important insights about the routes to take for the establishment of reliable drug quantification assays and highlighted current issues. In the present article/white paper, the different steps and results of the development for these two methods are presented. The different conclusions that were drawn from and for method development are reviewed. Additionally, the currently unsolved problems related to drug quantification with MALDI-MS are illustrated, and methods and preliminary results for addressing these challenges are presented. Finally, the different parameters for method development and perspectives about future developments that would be necessary for drug quantification assays using MALDI-MS are summarized.

## 2. Results

### 2.1. Tissue Sections

Using recent developments performed with an MALDI-ion mobility-tandem mass spectrometry (IM-MS/MS) assay for the quantification of the model drug MBZ in tissue sections applying a profiling method (manual deposition of the MALDI matrix, with no information on the spatial distribution) [11] can help illustrate current methodological challenges and new developments for addressing them.

#### 2.1.1. Ion-Mobility for Post-Acquisition Signal Filtering

One critical issue of MALDI-MS assays for drug quantification is the lack of analytical specificity and thus sensitivity for the quantification of drugs in complex mixtures. Using the MALDI matrix 2,5-DHB, which is known to be versatile for the ionization of small compounds, including the model drug MBZ, we could evaluate the usefulness of ion mobility spectrometry (IMS) as a post-ionization separation method to increase the selectivity of analyses after data extraction [11]. In this study, developments started with a standard MALDI-MS assay to quantify the intact compound in the tissue sections, and the MS method was gradually modified by adding IM separation and MS/MS fragmentation to finally reach a multiple reaction monitoring (MRM)-like method based on the monitoring of a fragment ion with an *m/z* specific to the compound of interest. In addition, we also optimized data analysis. Overall, the following conclusions were drawn:

1. Traveling wave (TW)IM-MS increased the resolution and overall intensity of MS signals;
2. When used for post-acquisition data filtering, IM-MS yielded a higher selectivity and thus resulted in a higher sensitivity;
3. Besides confirming the identity of the compound of interest, MS/MS diluted background signals from the MALDI matrix and endogenous interfering compounds because fragments of all compounds could be analyzed through a broader mass range. Therefore, fragments of the drug of interest and its internal standard (IS) could clearly be distinguished from fragments of endogenous compounds;
4. The use of an LC-like data treatment method based on an estimation of the area-under-the-curve (AUC) of the extracted IM peak (“MobA”) yielded more reliable and reproducible quantification results.

Overall, the developments in this study enabled a 200-fold reduction of the lower limit of quantification (LLOQ) for MBZ from 1000 to 5 ng/g.

#### 2.1.2. Method Pre-Validation and Quantification in Dosed Tissues

Based on guidelines established by the FDA and EMA [12,13], we demonstrated the possibility of validating the method using one validation-like batch with a full calibration curve and quality control samples (QCs) for accuracy and precision assessment. The different QCs consisted of LLOQ, low QC ((LQC) = 3 × LLOQ), middle QC ((MQC) = 30–50% of the calibration range), and high QC ((HQC) = at least 75% of upper limit of quantification (ULOQ)). The linearity of the method was proven with a determination coefficient of r^2^ = 0.9974 using a linear regression with 1/x weighting. Accuracies and precisions of all calibration standard samples (CALs) were below or within the limits of ±15% bias and 15% coefficient of variation (CV), while LQC, MQC, and HQC were accepted with a within-batch accuracy and precision within or below the ±15% bias and 15% CV limits. However, despite a within-batch precision below the 20% CV limit for the LLOQ (13.0% CV), the LLOQ was outside the accuracy criteria defined by the FDA and EMA (accuracy within ±20% bias), with a within-batch accuracy of 27% bias. One dosed tissue was also quantified and the results were compared with LC-ESI-MS/MS quantification data. The MALDI-IM-MS/MS assay yielded comparable results to the LC-ESI-MS/MS method, with only 19.4% deviation to the mean between the two methods (guidelines recommend a maximum of 20% deviation to the mean between comparison methods).

Further developments would be necessary to fully validate the method. This would necessitate using tissue sections from different animals, in order to assess the specificity of the method, as well as the reproducible matrix effect and analyte recovery between biological replicates across the full calibration range. Due to the large number of tissue sections from one individual source and from several different sources required to produce the CALs and QC samples and a full validation, it might be necessary to search for an alternative surrogate biological matrix. In such a case, the alternative biological matrix should also be validated, i.e., it should be proven during the validation that the obtained results are similar to those obtained with the biological matrix to quantify. Regarding data interpretation, another weighting method, such as 1/x^2^, might be necessary for conducting a full validation, in order to correct for signal variability and consequently high accuracy values at low concentrations (see below, Table 1).

The distinctive advantages of this method were that it was much faster than LC-ESI-MS/MS and necessitated a lower sample input. However, since the crystals of 2,5-DHB are very heterogeneous over the surface, it was necessary to manually control where the laser was directed (manual motion) in order to target the MALDI matrix crystals and ensure a stable signal during the analysis. Further developments are thus necessary to automatize MALDI-MS acquisition.

### 2.2. Plasma

This section explains the detailed steps of development of a recently established MALDI-MS assay for the quantification of OSN in plasma, in order to illustrate the drawbacks in sample preparation and successful solutions [9].

#### 2.2.1. Determination of the MS Mode

Based on the experience gathered from IM and MS/MS (Section 2.1.1), we hypothesized that a MALDI-IM-MS/MS approach would be necessary for drug quantification from plasma. In this study, 2,5-DHB was used as the standard MALDI matrix because of its versatility for the ionization of small compounds. MS/MS analyses for OSN revealed that the highest intensities were found for fragments with a mass-to-charge ratio (*m*/*z*) of 185.10 for OSN and *m*/*z* 189.12 for its IS—[^13^C,^2^H_3_]-OSN. These fragments were not previously described using ESI [9], and the fragmentation process still remains to be elucidated.

**Figure 1:**
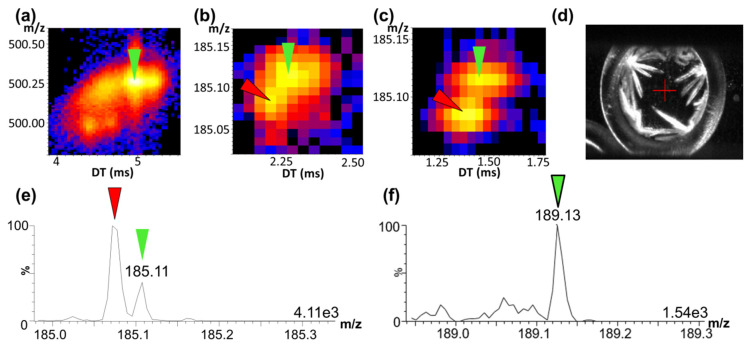
Two-dimensional mass-to-charge ratio (*m*/*z*) vs. drift time (DT) maps of native osimertinib (OSN, *m*/*z* 500.25) directly spiked in plasma (**a**), the major fragment of OSN (*m*/*z* 185.10) using a single-ion reflection mode (sensitivity mode) (**b**), and a two-ion reflection mode (resolution mode) (**c**). Aspect of 2,5 dihydroxybenzoic acid (2,5-DHB) crystals after direct spiking and crystallization from untreated plasma (**d**). Mass spectra of OSN spiked at 100 ng/mL (**e**) and its internal standard [^13^C,^2^H_3_]-OSN (**f**) in untreated plasma with 2,5-DHB. The maximum intensities of the mass spectra are indicated at the bottom right of the spectra. OSN signals are marked with green arrows, internal standard signals with green arrows with a black border, and interfering ion signals with red arrows.

An initial method consisting of mixing the MALDI matrix 2,5-DHB with native plasma was tested, as described in Section 4.2. As observed in tissue sections, the molecular complexity of the biological matrix did not allow OSN to be distinguished from the interfering endogenous compounds. This confirmed the absolute necessity of using MS/MS-based methods to monitor a specific fragment of OSN, instead of the intact compound (Figure 1a). The next results of MALDI-IM-MS/MS indicated that the use of MS/MS combined with IM could not distinguish the major OSN fragment of interest from an interfering ion fragment and that a higher mass resolution was necessary to compensate for the lack of resolution of IM (Figure 1b). For this, the initial method with a single ion-reflexion step in the time-of-flight tube of the instrument (sensitivity mode) was converted into a method with two ion-reflexion steps (resolution mode) to achieve a higher mass resolution and to be able to resolve the signals of the OSN fragment and the interfering ion (Figure 1c,e) [9]. Although the sensitivity mode should give a lower LLOQ than the resolution mode for the quantification, the gain of resolution helped to effectively detect the signal of interest, even when its intensity was lower than the intensity of the interfering signal, thus enabling a better sensitivity to be achieved for our assay by using the resolution mode. The IS displayed no interferences (Figure 1f).

#### 2.2.2. Sample Preparation Developments for Automatic Acquisition

The main goal of method development was to provide an assay that would be substantially faster than other MS methods and include automatic acquisition. The method consisting of directly mixing the MALDI matrix with untreated plasma led to the formation of low-density white crystals (Figure 1d). It was demonstrated that the high viscosity of plasma did not allow for the proper crystallization of 2,5-DHB. The 2,5-DHB crystal morphology after the addition of untreated plasma did not allow a constant signal to be obtained using an automatic motion. Different motion patterns were designed and tested to allow the laser to better reach the 2,5 DHB crystal “sweet spots”, but none provided any significant improvement.

We aimed to find a workaround to render 2,5-DHB suitable for high throughput automated quantification in plasma that could be universally applied to other MALDI matrices displaying heterogeneous crystallization, and using any motion pattern [9]. Therefore, a strategy of liquid-liquid extraction (LLE) coupled to sample deposition on pre-spotted 2,5-DHB was adopted, with a triple effect:

1. Drug enrichment from the sample;
2. Reduction of the sample viscosity and molecular complexity;
3. Re-crystallization of heterogeneous 2,5-DHB crystals to yield a homogenous layer leading to higher and more stable signals over the time of analysis.

Despite the resulting very low viscosity of LLE extracts to be deposited on the pre-spotted 2,5-DHB matrix, solution spreading was limited to the surface of the spots thanks to the presence of high-density 2,5-DHB at the edge of pre-spotted positions. Using this approach, an LLOQ of 5 ng/mL was reached for OSN [9].

#### 2.2.3. Method Validation

The optimized method was fully validated according to the guidelines established by regulatory authorities (FDA and EMA), with some adaptations to fit the specificities of the MALDI-IM-MS/MS assay [9]. Because sample preparation relied on LLE, all parameters for validation could be verified, including recovery after extraction. Contrary to sample preparation for LC-MS, no further sample handling is performed after LLE besides sample deposition on 2,5-DHB-prespotted targets. Indeed, using regular LC-MS/MS sample preparation, LLE extracts are dried and resolubilized with the appropriate solvent for the analysis [14]. Tert-butyl methyl ether (TBME) is indeed frequently used for LLE, but not compatible with LC-ESI-MS/MS analyses. For the MALDI-IM-MS/MS assay, regular QCs (low, medium, and high) were prepared, as well as CALs, and dedicated QCs for recovery and matrix effect reproducibility assessment in biological matrices from different sources. However, using our approach, any handling of LLE extracts led to a dilution effect. Therefore, a QC DIL series was created, consisting of QCs diluted with a blank solvent mimicking the solvent composition of the LLE upper layer (UL) also called the “blank UL mix” [9]. The blank UL mix was also used to create the QC for recovery (QC REC) and matrix effect assessment (QC MAT) that were compared to the QC DIL series. Moreover, although the developed approach allowed a more stable signal thanks to the matrix recrystallization, the MALDI process still showed signal variability between analytical replicates due to the surface heterogeneities that can still be present after optimization. For the QC REC series, it was then important to spike the IS before the extraction for further normalization and correction of analytical variations. Recovery was then calculated from the normalized response of the OSN fragment. Although it required additional steps for sample preparation, we proved that full method validation was possible. As for the MBZ assay in tissue sections (Section 2.1), several options were tested for data extraction (i.e., the use of extracted mass signal intensities—MassI—or use of the AUC of the extracted mobility peaks—MobA) and regression model (i.e., weighting parameter of linear regression). Only the data extraction method mimicking the LC data extraction process (i.e., MobA) and correcting signal variations at low concentrations (i.e., 1/x^2^ weighting of the regression model) led to successful method validation. The comparison of the validation attempts using the different data extraction processes and weighting approaches for linear regression computation are summarized in Table 1.

In summary, the assay characteristics of this method confirm that MALDI is a valuable ion source for the development of rapid, sensitive, and fully validated methods for drug quantification in plasma, suggesting that MALDI should be considered within the panel of existing MS-based approaches for this application. It also illustrated that the well-known issue of crystal heterogeneity of a large panel of MALDI matrices can be solved in some cases using the combination of LLE and matrix re-crystallization. This specific development largely extends the field of possibilities toward the development of MALDI-MS methods for the quantification of drugs in plasma, when optimal ionization of the compounds of interest can only be achieved with MALDI matrices that crystallize heterogeneously.

### 2.3. Further Developments for Automatic MS Acquisition in Tissue Analyses

Here, the aim is to focus on the development and optimization of bioanalytical assays for the rapid quantification of drugs in tissues when a high spatial resolution is not needed. Therefore, the following development paths do not concern MALDI-MS in imaging mode, but only that in profiling mode. As mentioned in Section 2.1, the developed MALDI-IM-MS/MS assay for rapid profiling in tissue using 2,5-DHB was competitive compared to LC-ESI-MS/MS in terms of the rapidity, and a strategy for automatic MALDI-MS acquisition remains to be developed. Currently, in order to target 2,5-DHB crystals that provide optimal MS signals, previous experience with MALDI-MS and an experienced observer are necessary to manually control the sample motion and perform the analyses. The morphology of the matrix crystals does not allow a constant signal to be employed when automatic motion patterns are used during acquisition, possibly hampering sensitive and reliable quantification.

**Figure 2:**
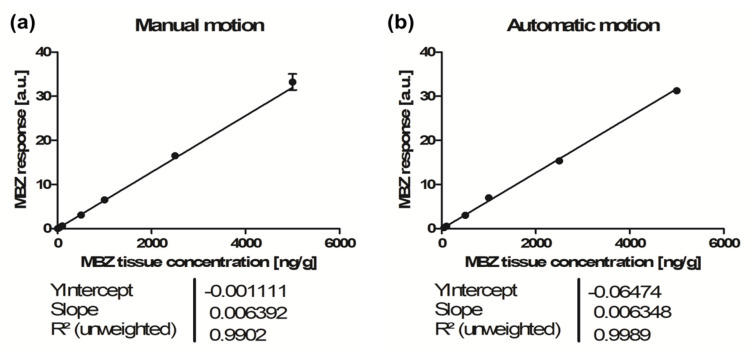
Calibration curves and linear regression models (1/x^2^ weighting) obtained from matrix-assisted laser desorption/ionization—ion mobility—tandem mass spectrometry (MALDI-IM-MS/MS) acquisition for mebendazole (MBZ) quantification in tissue sections using 2,5-dihydroxybenzoic acid (2,5-DHB) and manual motion (**a**) or automatic motion (**b**).

We verified this statement by attempting to construct a calibration curve using automatic motion without further 2,5-DHB crystal optimization for analyses. Figure 2 displays the calibration curves of manual and automatic motion methods and Table 2 lists the results of replicate analyses. With manual motion, all eight calibration levels from 5 to 5000 ng/g were accepted with ≥50% of replicates within the ± 15% bias accuracy limits, and overall, 72% of the total replicates were accepted (Table 2). The calibration curve was computed using a linear regression model with 1/x^2^ weighting, yielding a determination coefficient of r^2^ = 0.9902, with an LLOQ at 5 ng/g (Figure 2a). With automatic motion, when using the same criteria, only five calibration levels out of eight were accepted, and overall, only 44% of the total replicates were accepted (Table 2). Over the accepted calibration range from 50 to 5000 ng/g, the calibration curve yielded a determination coefficient of r^2^ = 0.9989 (Figure 2b) and the obtained LLOQ was 10-fold higher than that for the manual motion (Table 2).

This test confirmed the current necessity to perform manual MALDI-MS acquisitions to obtain reproducible results and meet the quantification standards of regulatory guidelines. Further developments are thus necessary to implement automatic motion for MALDI-MS analyses in profiling mode. In the following, we approached two options to permit rapid automatic motion using the method: (i) Using MALDI matrices displaying homogenous crystallization, and (ii) using TBME as a solvent for 2,5-DHB solubilization/recrystallization.

#### 2.3.1. Automation of MS Acquisition Using Alternative MALDI Matrices

**Figure 3:**
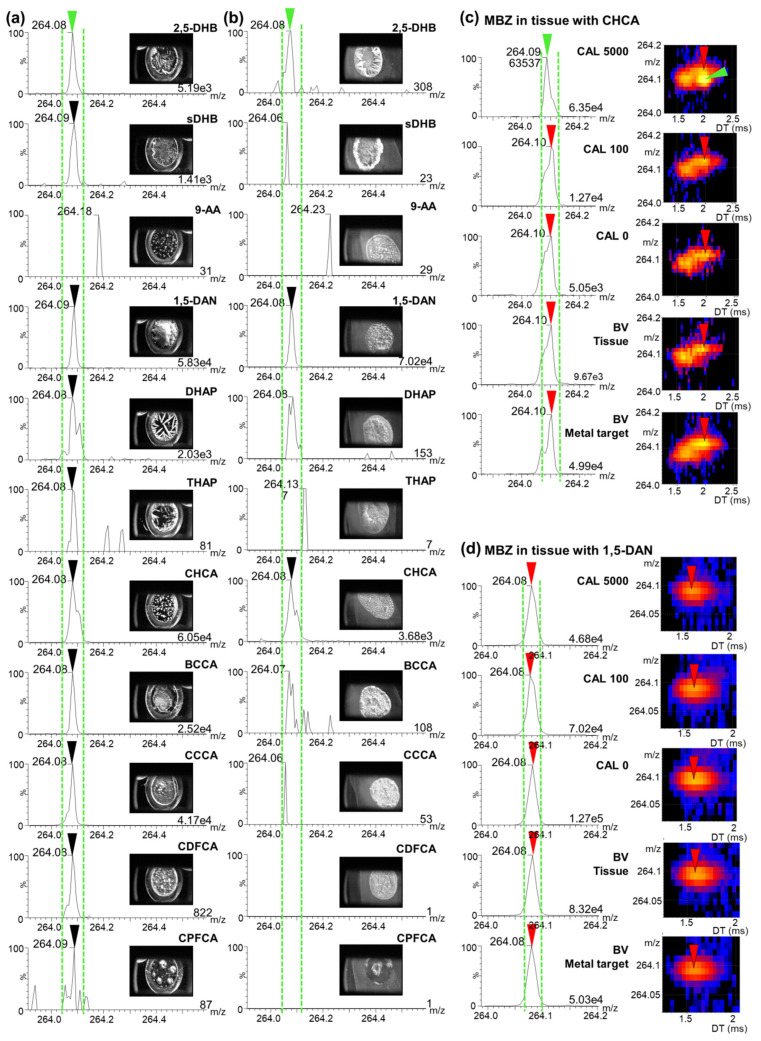
(**a**) Mass spectra of mebendazole (MBZ) calibration standard solution at 100 ng/g (CAL 100) were spotted on a matrix-assisted laser desorption/ionization (MALDI) metal target covered with different MALDI matrices. Spectra were acquired with automatic motion, showing the signal in the mass range of the fragment of interest of MBZ (green and black arrows, targeted mass window between green dotted lines). (**b**) Mass spectra of CAL 100 spiked on tissue sections with different MALDI matrices, showing the signal in the mass range of the fragment of interest of MBZ (green and black arrows, targeted mass window between green dotted lines) (**c**) Mass spectra and two-dimension (2D) mobility maps of blind value (BV), CAL 0, CAL 100, and CAL 5000 in α-cyano-4-hydroxycinnamic acid (CHCA) acquired on tissue, showing the signal of interfering fragments (red arrows) and the fragment of interest of MBZ at the highest calibration point (green arrow). (**d**) Mass spectra and 2D mobility maps of BV, CAL 0, CAL 100, and CAL 5000 in 1,5-diaminonaphtalen (1,5-DAN) acquired on tissue, showing the signal of interfering fragments (red arrows). Maximum intensities are given at the bottom right of each spectrum in arbitrary units. 9-AA, 9-aminoacridine; BCCA, 4-bromo-α-cyanocinnamic acid; CCCA, 4-chloro-α-cyanocinnamic acid; CDFCA, α-cyano-2,4-difluorocinnamic acid; CPFCA, α-cyano-2,3,4,5,6-pentafluorocinnamic acid; DHAP, dihydroxyacetophenone; 2,5-DHB, 2,5,-dihydroxybenzoic acid; sDHB, super-DHB; THAP, trihydroxyacetophenone.

One solution for obtaining stable and high MS signals consists of using alternative matrices for the analyses that yield homogenous crystallization after manual deposition on the surface to analyze. In this context, it is necessary to verify the potential of the MALDI matrix to ionize the compound of interest before performing automated analyses. Considering this, we tested ten additional MALDI matrices for their crystal morphology and their potential to ionize MBZ in the absence of a biological matrix on a MALDI metal target or spiked on the tissue section. In the panel of matrices that were selected, the α-cyano-4-hydroxycinnamic acid (CHCA) matrix was used due to its crystallization, which is known to be deposited more homogenously than 2,5-DHB on the deposition surface. We also tested second generation cinnamic acid (CA) MALDI matrices, namely 4-bromo-α-cyanocinnamic acid (BCCA); 4-chloro-α-cyanocinnamic acid (CCCA), α-cyano-2,4-difluorocinnamic acid (CDFCA), and α-cyano-2,3,4,5,6-pentafluorocinnamic acid (CPFCA). Additionally, we tested other MALDI matrices for small molecules, including 9-aminoacridine (9-AA), 1,5-diaminonaphtalen (1,5-DAN), dihydroxyacetophenone (DHAP), super-DHB (sDHB), and trihydroxyacetophenone (THAP). From the tests on MBZ in the absence of a biological matrix, ten matrices displayed a signal at the mass of MBZ, namely 2,5-DHB, sDHB, 1,5-DAN, DHAP, THAP, CHCA, BCCA, CCCA, CDFCA, and CPFCA, and 1,5-DAN, CHCA, CCCA, and DFCCA created a homogenous layer of crystals (Figure 3a). However, when applied on tissue, only 1,5-DAN and CHCA appeared to be potential candidates in terms of the ionization of MBZ and crystal homogeneity (Figure 3b). This revealed how important it is to take into account the biological matrix and thoroughly test MALDI matrix candidates during method development. In complex mixtures, a strong ion suppression effect takes place, which can lead to the complete extinction of the signal. After further examination of the IM-MS profile, strong interference could be observed in the vicinity of the MBZ signal using CHCA (Figure 3c). A calibration curve was attempted and additional strong interference was present in CAL 0 (blank biological matrix only spiked with the IS) and blind values (blank biological matrix), thus leading to an LLOQ of 5000 ng/g. The IM-MS profile of MBZ in 1,5-DAN also revealed the presence of interference in the vicinity of the MBZ fragment, as shown in Figure 3d for the MS spectra of blind values and CAL 0 samples.

These tests again illustrated the relative versatility of 2,5-DHB for the ionization of small compounds and the necessity to develop methods to obtain the homogeneous crystallization of 2,5-DHB from tissues.

#### 2.3.2. Automation of MALDI-MS Analyses on Tissue Sections Using 2,5-DHB as a MALDI Matrix

**Figure 4:**
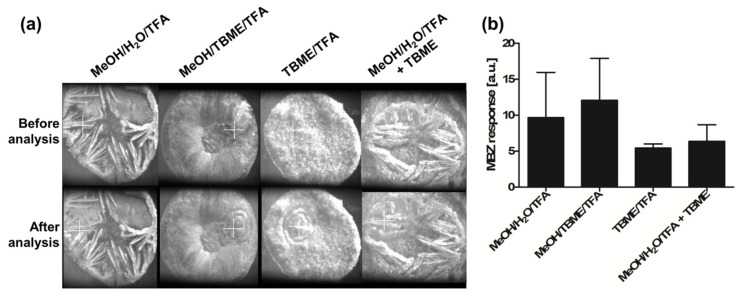
(**a**) Crystal morphology of 2,5-dihydroxybenzoic acid (2,5-DHB) in MeOH/H_2_O/trifluoroacetic acid (TFA) 50:50:0.1 (*v*/*v*/*v*), MeOH/tert-butyl methyl ether (TBME)/TFA 50:50:0.1 (*v*/*v*/*v*), TBME/TFA 100:0.1 (*v*/*v*), and MeOH/H_2_O/TFA 50:50:0.1 (*v*/*v*/*v*) followed by resolubilization in TBME on tissue. (**b**) Histogram showing the mebendazole (MBZ) response from dosed tissues and the variability between replicates, depending on the 2,5-DHB solvent composition. Triplicates were used for these analyses, and error bars represent the standard deviation.

The experience from former developments for plasma analyses [9] and new experiences in tissue section analyses indicated that 2,5-DHB appears to remain one of the most versatile MALDI matrices for the ionization of small compounds and that solutions can be found to overcome signal variability problems due to heterogeneous crystallization. In some applications for drug quantification, it is likely that 2,5-DHB may be the single option of the MALDI matrix for ionization. Here, we aimed at exploiting the potential of TBME for drug extraction and 2,5-DHB solubilization as a further option for quantification in tissue sections. Several sample preparation options were tested for the extraction of MBZ from dosed tissues (Section 4.1), as illustrated in Figure 4. 2,5-DHB was either solubilized as usual with acidified aqueous MeOH (MeOH/H_2_O/trifluoroacetic acid (TFA) 50:50:0.1 (*v*/*v*/*v*)), with TBME mixed with acidified MeOH (MeOH/TBME/TFA 50:50:0.1 (*v*/*v*/*v*)), or with acidified TBME (TBME/TFA 100:0.1 (*v*/*v*)). A method adapted from the sample preparation method for plasma samples was also attempted, consisting of first depositing 2,5-DHB in MeOH/H_2_O/TFA 50:50:0.1 (*v*/*v*/*v*) spiked with the reference standard and the IS, and resolubilizing 2,5-DHB crystals with TBME (double deposition method) (Figure 4a). Only-2,5-DHB in acidified TBME displayed fully homogenous crystallization over the deposited area, leaving no blank space for the analysis. The double deposition method only allowed a small portion of the 2,5-DHB crystals to be solubilized as the crystals of 2,5-DHB mixed with standards and IS did not allow TBME to be retained in the same way as with pure 2,5-DHB crystals in the assay developed for plasma.

The results indicated that lower responses were obtained using 2,5-DHB in TBME/TFA 100:0.1 (*v*/*v*); however, the obtained values were more reproducible than when using 2,5-DHB in MeOH/TBME/TFA 50:50:0.1 (*v*/*v*/*v*) or in MeOH/H_2_O/TFA 50:50:0.1. The double deposition method led to more variability between replicates than with acidified TBME alone (Figure 4b).

**Figure 5:**
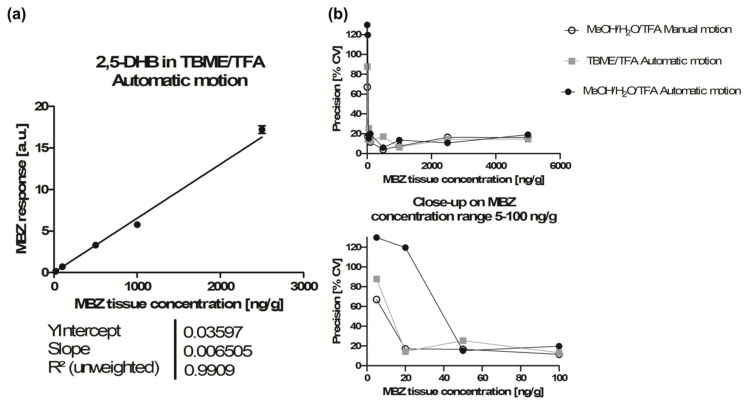
(**a**) Calibration curve obtained for mebendazole (MBZ) quantification in tissue using the MALDI matrix 2,5-dihydroxybenzoic acid (2,5-DHB) dissolved in tert-butyl methyl ether (TBME)/trifluoroacetic acid (TFA) 100:0.1 (*v*/*v*). (**b**) Precision graphs for calibration points using 2,5-DHB in MeOH/H_2_O/TFA 50:50:0.1 (*v*/*v*/*v*) and manual or automatic motion, or using 2,5-DHB in TBME/TFA 100:0.1 (*v*/*v*).

A calibration curve was performed in order to test the performance of the acidified TBME method for MBZ quantification in tissue following the regulatory guidelines. An LLOQ of 20 ng/g was reached, demonstrating that the automatic motion approach used with 2,5-DHB in TBME/TFA 100:0.1 (*v*/*v*) was more sensitive than that with 2,5-DHB in MeOH/H_2_O/TFA 50:50:0.1 (*v*/*v*/*v*) (Table 2 and Table 3). An r^2^ = 0.9909 was obtained (Figure 5a) and the precision values obtained for low concentrations were better than those for 2,5-DHB in MeOH/H_2_O/TFA 50:50:0.1 (Figure 5b). However, two calibrations levels were excluded from the curve and overall, more than 25% of all the calibration point replicates were excluded (Table 2).

It is noteworthy that the responses obtained for the calibration curve were found in similar ranges using 2,5-DHB in TBME/TFA or in MeOH/H_2_O/TFA (Figure 2 and Figure 5). However, although the responses obtained for the dosed tissue were comparable between replicates, the mean response was about 50% lower using 2,5-DHB in TBME/TFA compared to 2,5-DHB in MeOH/H_2_O/TFA. In order to verify which value was the most accurate, we aimed to quantify MBZ from the same tissue using the two approaches for solubilization of the MALDI matrix, and using a fully validated LC-ESI-MS/MS approach in parallel [11]. The LC-ESI-MS/MS results revealed an MBZ concentration of 1780 ng/g; the MALDI-MS/MS results using 2,5-DHB in MeOH/H_2_O/TFA, 1511 ng/g; and the MALDI-MS/MS results using TBME/TFA, 828 ng/g. Therefore, when using TBME/TFA as a solvent for 2,5-DHB, the quantities of MBZ in dosed tissues were largely underestimated. This could be explained by a lower extraction power of MBZ when using TBME compared to MeOH/H_2_O. This observation is crucial for the development of MALDI-MS for drug quantification from tissue sections. Although the establishment of calibration curves could provide satisfactory values in terms of precision due to homogenous crystallization, it is important to verify that the solvent used for matrix solubilization permits the adequate extraction of the drug from dosed tissues. Drugs from dosed tissues and IS must be well-mixed together in order to permit an optimal correlation with the calibration curve. Without a proper extraction of the drugs from dosed tissues, the IS deposited on top of the tissue sections may be more accessible than the drug, thus leading to an overcorrection during the normalization.

These latter developments indicate that using alternative solvents for 2,5-DHB dissolution is a valuable way to enable automation for even more rapid drug quantification in situ. However, great care has to be taken to ensure the optimal extraction of drugs from tissue sections and avoid underestimation or overestimation of the true content. Further developments are necessary to find adequate solvents permitting both optimal 2,5-DHB solubilization and optimal extraction of drugs from tissues. This also highlights the high importance of using LC-MS/MS in parallel with MALDI-MS method development for the cross validation of quantification results.

## 3. Discussion

Theoretically, MALDI-MS allows for direct and extremely rapid analyses compared to LC-MS approaches for solid or solidified samples.

In the present white paper, we aimed to review our experience with past developments that led to the successful establishment of pre-validated or fully validated MALDI-MS assays for drug quantification in liquid (plasma) or solid samples (tissue sections). In this context, it was possible to highlight the different hurdles that can be encountered on the way to the successful development of these assays, as well as some of the remaining issues. Additional tests and developments were performed in order to generate new solutions for issues such as the automation of analyses in tissue sections. More importantly, the wealth of experiences gained from these experiments, together with earlier reports, allows us to draw important conclusions regarding the establishment of such rapid quantitative assays, from the selection of the MALDI matrix to strategies of data extraction.

### 3.1. Biological Matrix

The nature of the biological matrix influences the suitable options for sample preparation. Liquid samples allow the use of LLE and LLE extracts that can be analyzed directly using MALDI without compatibility problems with the extraction solvents. Therefore, using LLE for plasma samples does not alter the advantage of the overall rapidity of the MALDI-MS-based workflow because sample drying and resuspension steps are not essential for sample preparation. Solid samples such as tissue sections allow for direct analyses in a histological context, without an extraction step. However, the economy of time comes at the expense of the quality of MALDI crystals due to the high sample complexity. Therefore, methods have to be adapted in order to permit both optimal extraction of drugs from tissue sections and optimal desorption/ionization from MALDI crystals.

### 3.2. MALDI Matrix Selection

Rapid MALDI-MS quantification requires straightforward sample handling and therefore straightforward MALDI matrix deposition. However, each MALDI matrix presents a different crystal morphology, especially when deposited manually. Heterogeneous crystallization hampers signal stability when automatic motion is used during MS acquisition, and eventually, also the establishment of quantification assays, as demonstrated in this white paper. Additionally, our experience illustrates that MALDI matrices displaying homogeneous crystallization do not necessarily ionize the compounds of interest and can create important chemical interferences. 2,5-DHB seems to remain the most versatile MALDI matrix for the ionization of small molecule drugs, but presents heterogeneous, spiky crystallization that does not allow for sensitive and reproducible analyses. However, workarounds can be found to recrystallize 2,5-DHB into homogenous layers, e.g., using LLE extracts with TBME as extraction solvent. This approach can successfully be applied and validated following regulatory guidelines for drug quantification in biological fluids. Our experience indicates that TBME can be used for the solubilization of 2,5-DHB in order to obtain homogenous crystallization on tissue after manual deposition. The resulting crystals did not allow optimal desorption/ionization of the compounds of interest, but they allowed more stable signals to be obtained, which increased the precision and accuracy. However, our results demonstrated that, applied on dried tissue, TBME apparently yielded lower extraction compared to MeOH/H_2_O solvent. This led to a large underestimation of the MBZ quantities in tissue sections. It is noteworthy that this underestimation could not be detected without the previous knowledge of quantities from LC-MS data. Therefore, for the development of MALDI-MS methods for drug quantification, LC-MS remains the gold standard for cross-validation (see Section 3.6). Future developments could consist of screening solvents that would allow for the solubilization of MALDI matrices, their homogenous crystallization on tissue sections, and the optimal extraction of targeted drugs. Possible future options also consist of using ionic liquid MALDI matrices for homogenous deposition. However, the potential of a solid MALDI matrix to accumulate energy from the laser and its ability to produce ions cannot be directly translated into ionic liquid MALDI matrices. An additional possibility would be to develop methods for the automatized morphological detection of MALDI crystals, which would likely yield optimal desorption/ionization. However, this detection should be performed rapidly, in order to keep the MALDI-MS method as a whole time-efficient.

### 3.3. Sample Preparation

As illustrated by our observations with plasma, the strategy for sample preparation has to be chosen according to the selected MALDI matrix. First, mixing 2,5-DHB directly with plasma hampered the formation of crystals. Additional tests with other MALDI matrices indicated that this was a general effect that can also lead to total signal extinction in some circumstances (data not shown). After LLE, depositing the low-viscous extract directly on pre-spotted 2,5-DHB was possible thanks to the heterogeneous morphology of 2,5-DHB crystals. The spiky crystals at the edge of the deposits prevented the spreading of TBME from the surface. Alternatively, using dedicated surfaces for sample deposition that would permit a homogenous crystallization of MALDI matrices (e.g., hydrophobic surface, Anchorchips) [15,16] would be another option for the development of MALDI-MS assays for drug quantification in biological fluids. Using solid samples such as tissue sections for direct analyses leaves fewer options for sample preparation. Workarounds such as adapting the solvent for solubilization of the MALDI matrix can be found (e.g., TBME, see Section 2.3.2) to obtain homogenous samples when matrices displaying heterogeneous crystals are used. However, it is important that homogenous crystals still optimally accumulate energy from the laser for proper desorption/ionization, and that the solvent used offers a proper extraction of drugs from dosed tissues. In the frame of our tests in tissues, to reach a rapid sample preparation process, manual deposition of the MALDI matrix was chosen. However, using spotters may also represent a valuable option for the localized automated deposition of picoliter/nanoliter volumes of solutions of standards and MALDI matrices [17]. Homogenous crystallization and reproducible depositions may be obtained with satisfactory time-efficiency.

Additionally, the concentration of the MALDI matrix and its ratio with the sample is highly important. Since only a minor fraction of ions are present in the MALDI plume during the regular MALDI process [18,19], it is important that ions of interest constitute a representative population in the MALDI plume, in order to eventually reach a satisfactory sensitivity for drug quantification. On the other hand, it is important to adapt the concentration of matrix to the complexity of the sample. Although different MALDI matrices are adapted to the ionization of different classes of compounds, these are not specific to a particular compound. It is therefore necessary to increase the chances of also ionizing the drugs of interest. For this, high concentrations of MALDI matrices might be necessary in complex mixtures. This latter point is thus also a crucial parameter to optimize, depending on the biological matrix to analyze. The concentration of the MALDI matrix also defines the crystallization homogeneity throughout the sample on the chosen surface. Our experience with analyses in plasma indicated that a concentration of 100 mg/mL of 2,5 DHB was optimal when 1 µL was deposited on classical metal targets for the creation of pre-spotted MALDI arrays [9]. The downstream deposition of LLE extracts from plasma created a homogenous layer of 2,5 DHB recrystallized with the sample.

### 3.4. Analysis

Irrespective of the instrumentation type, a major parameter for ion production is the fluence of the laser irradiating the sample. The intensity must not be too low to exceed the ionization threshold, while must also not be too high to avoid in-source fragmentations. Our experience with plasma analyses indicated that using too weak laser intensities resulted in a lower result reproducibility for measurements of low concentrations of standards. This led to the rejection of pre-analytical batches, according to regulatory guidelines (data not shown). Downstream, the type of MS analyzer is of major importance for the MALDI-MS assay for drug quantification. The analysis of complex mixtures can dramatically limit the assay specificity since the compounds of interest are directly analyzed together with endogenous compounds without separation prior to ionization. When using MS instrumentation with an insufficient resolution power, MS peaks can comprise isobaric ions. The large presence of these isobaric endogenous compounds often masks the drugs of interest at low concentrations. The analytical specificity can be increased using triple quadrupole instruments that allow for the precise selection of daughter ions for the further quantification of compound-specific transitions [7]. High resolution mass spectrometers allow for the discrimination of nearby isobaric compounds and therefore represent another valuable alternative for increasing the specificity for drug quantification in complex mixtures [10]. We recently demonstrated that IMS represented a very good alternative for increasing the analytical specificity for drug quantification thanks to gas-phase separation and post-acquisition signal filtering [11]. A higher IMS resolution power may even increase the separation of drugs from endogenous compounds. Using an alternative IMS setup such as trapped IMS (TIMS) [20] may also foster specificity during the analysis, by filtering ions of interest from interfering ions in the gas phase before MS separation in the analyzer. The combination of IMS with high resolution MS also represents an ultimate option for achieving ultra-high specificity analyses. In addition to the MS analytical process, MS ionization processes can also largely be improved using MALDI. This is exemplified through the development of laser-post-ionization (also called MALDI-2), which consists of irradiating the MALDI plume with a second laser in order to ionize compounds that are desorbed but not ionized after the first laser irradiation of the sample. MALDI-2 can increase the signal intensities of drugs from tissue sections by up to two orders of magnitude [19], and thus represents a promising option for increasing the assay sensitivity.

The characterization of parent and daughter ions using MS and MS/MS modes, respectively, is a parameter of interest using MALDI-MS. It is important to note that the chemical reactions occurring in the MALDI plume are more complex than those in the Taylor cone formed in ESI [1]. In the MALDI plume, reduction reactions often take place due to the presence of photoelectrons in the MALDI source. These photoelectrons can be generated by (i) photoionization of the MALDI matrix, or (ii) photoejection from the MALDI metal target induced by MALDI matrix, which can act as a proton conductor at the MALDI matrix–metal interface. These reactions can lead to analyte reduction with the formation of ion species other than [M + H]^+^ or [M − H]^−^ [1]. Additionally, although MALDI is considered as a soft-ionization ion source, in-plume charge-transfer reactions can lead to a significant degree of in-source decay (ISD), depending on the used MALDI matrix [1]. Possible fragmentations (ISD but also post-source decay (PSD)) have to be carefully monitored during method development. The proper understanding and prediction of complex ionization, redox, and fragmentation reactions occurring between MALDI matrices and drugs of interest during the MALDI process would greatly benefit to the improvement of the characterization of chemical species targeted in quantification assays. This would necessitate dedicated investigations of fundamental MALDI processes. Once these events are understood, strategies can be developed to enhance or inhibit chemical pathways of interest. For example, the combined use of reducing MALDI matrices and metallic targets might enhance the processes of analyte reduction, and vice versa. Such a strategy would lead to more homogenous populations of ions to focus on for quantification, and thus a higher sensitivity could be reached. In the absence of investigations of fundamental chemistry to precisely predict chemical reactions in the MALDI plume, it is necessary to choose ion species and fragments specific to the analyte by a data comparison of analyses of the compounds of interest and control analyses.

### 3.5. Data Extraction and Processing

In LC-MS/MS, the AUCs of peaks of interest are extracted from chromatograms for quantification purposes. Our experience with IMS indicates that using a similar data integration strategy as for LC-MS/MS (i.e., based on integrated AUCs from ions separated in the gas phase) increased the linearity of calibration curves and result reproducibility, and enabled bioanalytical method validation following regulatory guidelines [12,13]. This approach allows all ions in the *m*/*z* and mobility ranges corresponding to the compound of interest to be considered. Conversely, using maximum MS intensities or maximum mobility peak intensities for quantification only considers a small fraction of ions for calculations and can lead to variability between replicates. Therefore, the integration of extracted peak areas of the target compounds compared to the maximum peak intensity should be chosen for quantification whenever possible. Regarding regression model parameters, our experience indicates that models using 1/x^2^ weighting should be favored to correct MALDI-MS signal variations in the lower concentration range.

### 3.6. Cross Validation

As mentioned in Section 3.3, new developments in MALDI-MS can lead to artefacts in the quantification of drugs, especially when new solvents are used for the extraction of drugs from tissue sections. Overestimations and underestimations may not be detected without the use of parallel methods that reveal the true content of drugs in tissues. In this context, LC-MS is the current gold standard for the quantification of drugs in biological matrices, and should be considered as the method of choice for confirming MALDI-MS-based drug quantification, especially when developing new methods.

All these elements reveal the complexity of establishing MALDI-MS assays for drug quantification validated according to regulatory guidelines. Several parameters interlock together and the failure to satisfy one of these can hamper the whole workflow. The present results also demonstrate that the success of fully validating a method depends on parameters that have to be defined from initial steps of method development. However, and despite the difficulty of establishing an assay, MALDI-MS offers an incomparable time efficiency for therapeutic drug monitoring.

## 4. Materials and Methods

All materials and methods were formerly described in the upon-mentioned studies [9,11].

The analyses were performed using a Synapt G2-Si instrument (Waters Corp, Milford, NH, USA) consisting of an orthogonal acceleration (oa)-quadrupole (Q)-IMS-time-of-flight (TOF) spectrometer equipped with a MALDI source and controlled under MassLynx V4.1 (Waters). The instrument was used in resolution mode (“W” mode). A previously described IM-MS/MS method (“Method 4”) was tuned as follows:

*Ion source*: Laser intensity: 300 arbitrary units (a.u.), motion mode: spiral.

*Cooling gas*: 10 mL/min, trap gas: 2 mL/min, helium cell: 180 mL/min, IMS gas: 90 mL/min. Trap collision energy: 32 V. Transfer collision energy was off for all methods.

*Stepwave (SW) parameters.* SW1 wave velocity: 300 m/s, wave height: 15 V. SW2 wave velocity: 300 m/s, wave height: 15 V. SW2 DC offset: 25 V, differential aperture 1: 3 V, differential aperture 2: 0 V. Source ion guide: wave velocity: 300 m/s, wave height: 1 V. RF voltages: SW 300 V, ion guide: 350 V.

*Quadrupole parameters.* MS/MS mode was used with the targeted nominal mass of the compound of interest. The quadrupole low mass (LM) resolution was set to 4.4 a.u.

*TriWaveDC parameters.* Trap DC entrance: 3 V, bias: 45 V, trap DC: 0 V, exit: 0 V. IMS DC entrance: 20 V, helium cell DC: 50 V, helium exit: −20 V, bias: 3 V, exit: 0 V. Transfer DC: entrance 4 V, exit 15 V.

*TriWave parameters.* Nitrogen was used as IMS gas. Trap-wave velocity: 311 m/s, wave height: 4 V. IMS variable wave velocity from 1000 to 300 m/s ramping over a full IMS cycle, fixed wave height: 40 V. Transfer-wave velocity: 315 m/s.

The instrument was calibrated before every batch of analyses using a deposit of 20 mg/mL red phosphorus solution in MeOH/H_2_O/TFA 0.5: 0.5: 0.001 (*v*/*v*/*v*).

Additional sample preparations are described below.

### 4.1. Mebendazole Calibration Curves in Tissue Sections

#### 4.1.1. Chemical Reagents

MS-grade H_2_O and solvents were purchased from Biosolve Chimie SARL (Dieuze, France). MBZ and fenbendazole (FBZ) were purchased from Biozol (Eching, Germany). 2,5-DHB, TFA, and red phosphorus were purchased from Sigma-Aldrich (Darmstadt, Germany).

#### 4.1.2. Matrix Preparation

For MALDI-MS analyses, 2,5-DHB stock solutions of 200 mg/mL were prepared in MeOH/H_2_O/TFA (50:50:0.1 (*v*/*v*/*v*)) and TBME/TFA (100:0.1 (*v*/*v*)).

#### 4.1.3. Mebendazole Calibration Standard Preparation

For the samples using 2,5-DHB in acidified aqueous MeOH as the MALDI matrix, 3.41 µL of MBZ reference solution at the desired concentration was mixed with 43.18 μL of MeOH/H_2_O/TFA (50:50:0.1 (*v*/*v*/*v*)) and 3.41 μL of the IS (FBZ) solution at a fixed concentration. For the samples using 2,5-DHB in acidified TBME as the MALDI matrix, 2.3 μL of the MBZ reference solution at the desired concentration was mixed with 45.4 μL of TBME/TFA (100:0.1 (*v*/*v*)) and 2.3 μL of the IS solution at a fixed concentration. For CAL 0 samples, the volume of MBZ reference solution was replaced by the same volume of solvent, and for BV samples, 50 µL of solvent was taken. Subsequently, 30 μL of MALDI matrix solution in the required solvent was mixed with 10 μL of each calibration standard solution.

From each CAL mixed with the MALDI matrix, 1 μL of MeOH/H_2_O/TFA (50:50:0.1 (*v*/*v*/*v*)) and 1.5 μL of TBME/TFA (100:0.1 (*v*/*v*/*v*)) were deposited onto a tissue section, and up-and-down pipetting was performed three times, in order to extract endogenous compounds from the tissue in the deposition area. This resulted in virtual concentrations on tissue ranging from 5 to 5000 ng/g (CAL 5 to CAL 5000 samples, Table 2).

#### 4.1.4. Mass Spectrometry Parameters

For the analyses in resolution mode, the MALDI-MS method previously described as Method 4 in [11] was used. It was based on MS/MS fragmentation after selection by the quadrupole of the targeted parent ion, followed by IM separation.

#### 4.1.5. Data Processing for MALDI-MS Measurements

For IMS data extraction, MobA (use of the integrated AUC of the extracted mobility peaks) and MassI (use of *m*/*z* peak intensities) were used [11].

### 4.2. Osimertinib Screening in Untreated Plasma

#### 4.2.1. Chemical Reagents

OSN and [^13^C,^2^H_3_]-OSN were purchased from Alsachim (Illkirch, France).

#### 4.2.2. Matrix Preparation

A 2,5-DHB stock solution of 100 mg/mL was prepared in MeOH/H_2_O/TFA (50:50:0.1 (*v*/*v*/*v*)).

#### 4.2.3. Solution Preparation and Deposition on the MALDI Metal Target

A volume of 100 μL of plasma was mixed with 50 μL of the OSN reference solution at the desired concentration and 50 μL of the IS solution at a fixed concentration. In total, 50 μL of the mixture were mixed with 50 µL of the 2,5-DHB stock solution. The samples were vortexed and centrifuged for 1 min at 13,200× *g*. A total of one μL from the supernatant was then deposited on the MALDI metal target.

### 4.3. Testing of Different Matrices for Drug Quantification on Tissue Sections

#### 4.3.1. Chemical Reagents

The different MALDI matrices were purchased from Sigma-Aldrich (Darmstadt, Germany).

#### 4.3.2. Matrix Preparation

The following matrices were tested for the analysis of MBZ on the MALDI metal target and on tissue sections: 9-AA; BCCA; CCCA; CHCA; 1,5-DAN; DFCCA; DHAP; 2,5-DHB; sDHB; PFCCA; and THAP. 2,5-DHB and sDHB stock solutions of 200 mg/mL were prepared in MeOH/H_2_O/TFA (50:50:0.1 (*v*/*v*/*v*)). BCCA, CCCA, DFCCA, HCCA, and PFCCA stock solutions of 40 mg/mL were prepared in ACN/H_2_O/TFA (70:30:0.1 (*v*/*v*/*v*)). DHAP and THAP stock solutions of 60 mg/mL, and 9-AA and 1,5-DAN stock solutions of 40 mg/mL, were prepared in MeOH/H_2_O (70:30 (*v*/*v*)).

#### 4.3.3. Deposition on the MALDI Metal Target

For each MALDI matrix, 1 μL of the stock solution was pipetted on the MALDI metal target. After dryness, 1 μL of MBZ CAL 100 stock solution was deposited.

#### 4.3.4. Solution Preparation and Deposition on Tissue

For each solvent (Section 4.3.2), 43.18 μL of solvent was mixed with 3.41 μL of the MBZ reference solution at the desired concentration and 3.41 μL of the IS solution at a fixed concentration. Then, 30 μL of MALDI matrix solution was mixed with 10 μL of the prepared MBZ solution.

Finally, 1 μL of the MBZ solution mixed with the MALDI matrix was deposited onto a tissue section, and up-and-down pipetting was performed three times, in order to extract endogenous compounds from the tissue in the deposition area. All prepared tissue samples corresponded to CAL 100 MBZ calibration standards.

## Figures and Tables

**Table 1:** Summary of the accuracy and precision validation results obtained for the osimertinib matrix-assisted laser desorption/ionization—ion mobility—tandem mass spectrometry (MALDI-IM-MS/MS) quantification assay using two methods for data extraction: MassI (i.e., the use of extracted mass signal intensities) and MobA (i.e., use of the area-under-the-curve of the extracted mobility peaks). In addition, two weighting approaches were employed for linear regression computation: 1/x and 1/x^2^.

		MassI						MobA					
		Linear Regression 1/x Weighting			Linear Regression 1/x^2^ Weighting			Linear Regression 1/x Weighting			Linear Regression 1/x^2^ Weighting		
		Batch 1	Batch 2	Batch 3	Batch 1	Batch 2	Batch 3	Batch 1	Batch 2	Batch 3	Batch 1	Batch 2	Batch 3
Number of accepted calibration levels ^1^		5	5	5	7	6	7	7	6	7	7	8	8
% of accepted CALs ^1,2^		59%	63%	63%	75%	66%	78%	84%	75%	81%	84%	97%	88%
LLOQ accepted ^1,2^		🗶	🗶	🗶	✓	✓	✓	🗶	🗶	🗶	✓	✓	✓
ULOQ accepted ^1,2^		✓	✓	✓	✓	🗶	🗶	✓	✓	✓	✓	✓	✓
Determination coefficient r ^2^		0.9954	0.9980	0.9974	0.9894	0.9910	0.9907	0.9962	0.9993	0.9988	0.9915	0.9959	0.9971
Within-batch accuracies (% bias)	LLOQ	*194*	*385.5*	269.8	−4.7	11.1	−2.7	*64.2*	*70.6*	*32.3*	7.1	3.2	1.0
	LQC	*49.8*	*128.8*	73.0	−14.9	*25.8*	−8.2	14.8	*15.5*	5.1	2.7	−1.4	−2.8
	MQC	−15.0	11.4	−7.0	−11.5	*37.5*	6.8	−1.9	−4.6	−3.0	2.4	−0.7	0.6
	HQC	−6.5	4.3	−1.0	−0.9	*30.3*	16.0	−1.1	−3.8	−1.4	3.5	0.6	2.5
Within-batch precisions (% CV)	LLOQ	5.4	5.9	3.4	13.6	18.3	15.3	15.7	9.8	10.1	17.8	17.1	13.8
	LQC	5.2	5.9	4.7	9.8	12.2	10.5	4.5	5.8	7.3	5.3	9.4	8.2
	MQC	4.3	4.7	2.1	4.4	5.0	2.2	3.2	3.2	1.6	3.2	3.2	1.6
	HQC	5.4	5.9	3.8	5.5	5.7	3.9	4.5	5.0	1.5	4.5	5.0	1.5
Inter-batch accuracies (% bias)	LLOQ	*283.1*			0.8			*54.3*			2.3		
	LQC	*81.9*			−1.4			11.3			−0.6		
	MQC	−3.5			8.5			−3.2			0.8		
	HQC	−1-1			13.8			−2.1			2.2		
Inter-batch precisions (% CV)	LLOQ	*21.6*			16.7			16.5			15.0		
	LQC	10.0			*20.5*			7.2			7.8		
	MQC	12.3			*18.7*			2.9			3.0		
	HQC	6.7			12.2			4.0			4.0		
Overall percentage of QCS within the acceptance criteria ^2^		44%			64%			66%			89%		
Batch accepted ^1^		🗶	🗶	🗶	✓	🗶	🗶	🗶	🗶	🗶	✓	✓	✓
Method validated		🗶			🗶			🗶			✓		

^1^ A batch is only accepted if (i) at least six non-zero-calibration levels are accepted, (ii) both LLOQ and ULOQ levels are accepted (≥50% of replicates within the accuracy limits), (iii) all QC levels are accepted (≥50% of replicates within the accuracy and precision limits for within- and inter-batch values), and (iv) ≥75% of all calibration points are within the defined accuracy limits. ^2^ Acceptance criteria [12,13]: Accuracy should be within the ±15% bias limits or ±20% bias at the LLOQ, and precision values should be ≤15% coefficient of variation (CV) or ≤20% CV at the LLOQ. Values outside of the criteria are marked in cursive typing. CAL, calibration point; LOQ, limit of quantification (LLOQ, lower LOQ; ULOQ, upper LOQ); QC, quality control (LQC, low QC; MQC, middle QC; HQC, high QC).

**Table 2:** Results for the construction of the calibration curves for the quantification of mebendazole (MBZ) in tissue sections using 2,5-dihydroxybenzoic acid (2,5-DHB) in MeOH/H_2_O/trifluoroacetic acid (TFA) 50:50:0.1 (*v*/*v*/*v*) with manual or automatic motion.

	Manual Motion		Automatic Motion	
Calibration Levels	Number of Accepted Values (x/4) ^1^	Calibration Level Accepted ^2^	Number of Accepted Values (x/4) ^1^	Calibration Level Accepted ^2^
CAL 5	3/4	✓ (LLOQ)	0/4	🗶
CAL 20	2/4	✓	0/4	🗶
CAL 50	2/4	✓	3/4	✓ (LLOQ)
CAL 100	3/4	✓	2/4	✓
CAL 500	4/4	✓	4/4	✓
CAL 1000	4/4	✓	2/4	✓
CAL 2500	2/4	✓	1/4	🗶
CAL 5000	3/4	✓	2/4	✓

^1^ According to regulatory guidelines, accuracy within ±15% bias limits or ±20% bias at the LLOQ level. ^2^ According to regulatory guidelines, ≥50% accepted replicates per level. LLOQ, lower limit of quantification.

**Table 3:** Results for the creation of the calibration curve for the quantification of mebendazole (MBZ) in tissue sections using 2,5-dihydroxybenzoic acid (2,5-DHB) in tert-butyl methyl ether (TBME)/trifluoroacetic acid (TFA) 100:0.1 (*v*/*v*) with automatic motion.

Calibration Levels	Number of Accepted Values (x/4) ^1^	Calibration Level Accepted ^2^
CAL 5	1/4	🗶
CAL 20	2/4	✓ (LLOQ)
CAL 50	1/4	🗶
CAL 100	3/4	✓
CAL 500	2/4	✓
CAL 1000	2/4	✓
CAL 2500	3/4	✓
CAL 5000	1/4	🗶

^1^ According to regulatory guidelines, accuracy within ±15% bias limits or ±20% bias at the LLOQ level. ^2^ According to regulatory guidelines, ≥50% accepted replicates per level. LLOQ, lower limit of quantification.

## Data Availability

Not applicable.

## Sample Availability

Samples of the compounds mebendazole and osimertinib are available from the authors.
